# Occurrence of depression and assessment of functional capacity in patients with vascular diseases admitted to a Vascular Surgery Service

**DOI:** 10.1590/1677-5449.202300821

**Published:** 2023-12-18

**Authors:** José Aderval Aragão, Osmar Max Gonçalves Neves, Iapunira Catarina Sant’Anna Aragão, Felipe Matheus Sant’Anna Aragão, Bárbara Costa Lourenço, Luciano Conceição Porto, Pedro Henrique Adário Marassi, Francisco Prado Reis

**Affiliations:** 1 Universidade Federal de Sergipe - UFS, Aracaju, SE, Brasil.; 2 Fundação Beneficência Hospital Cirurgia - FBHC, Serviço de Cirurgia Vascular, Aracaju, SE, Brasil.; 3 Hospital Municipal Munir Rafful - HMMR, Volta Redonda, RJ, Brasil.; 4 Faculdade de Medicina de São José do Rio Preto - FAMERP, São José do Rio Preto, SP, Brasil.; 5 Faculdade de Ciências Médicas de Três Rios - FCM-TR, Três Rios, RJ, Brasil.; 6 Centro Universitário Tiradentes - UNIT, Maceió, AL, Brasil.; 7 Hospital Naval Marcílio Dias - HNMD, Rio de Janeiro, RJ, Brasil.; 8 Universidade Tiradentes - UNIT, Aracaju, SE, Brasil.; 9 Centro Universitário Alfredo Nasser - UNIFAN, Aparecida de Goiânia, GO, Brasil.

**Keywords:** vascular depression, vascular diseases, aptitude tests, frail elderly, activities of daily living

## Abstract

**Background:**

Vascular diseases are associated with significant sequelae and clinical repercussions for the lives of affected patients, which are more serious among the elderly. The consequences of vascular disease, such as limb loss, chronic pain, prolonged hospitalization, and polypharmacy, reduce these patients’ autonomy and independence, influencing their wellbeing and quality of life.

**Objectives:**

To determine the prevalence of depression and assess functional capacity in patients with vascular diseases admitted to a Vascular Surgery Service.

**Methods:**

This is a descriptive, cross-sectional study, carried out at the Vascular Surgery Service of a tertiary hospital with a non-random sample of patients selected consecutively. The geriatric depression scale short form (GDS-15) was used to assess depression and the Katz scale was used for functional assessment.

**Results:**

The prevalence of depression in these patients was 60.6%. Associations were observed between depression and consultation with a family doctor in the last 12 months, alcoholism, claudication, diabetes, and individuals who had had an amputation. Individuals’ Katz index functional capacity scores were significantly associated with sociodemographic variables, conditions related to vascular disease, and hospitalization.

**Conclusions:**

There was a high prevalence of depression in patients with vascular diseases admitted to a vascular surgery service and important reductions in functional capacity in some groups, such as individuals with low educational levels, those who had chronic pain in the lower limbs, patients with diabetes, and those who had had an amputation.

## INTRODUCTION

Vascular diseases (VDs) are caused by pathophysiologic changes in the arterial, venous, or lymphatic systems, such as reduced blood flow, compromising nutrient supply and causing ischemia or vascular stasis, depending on the vessel involved and tissue demand. Arterial involvement causes clinical manifestations, such as pain, trophic changes, pigmentation changes, cooling of limbs, and hair loss. Cases of venous involvement exhibit edema, hyperpigmentation, hyperhidrosis, eczema, ulcers, and dermatofibrosis. In cases with lymphatic involvement, pain and edema are more significant.^1^ Non-transmissible chronic diseases, such as diabetes, arterial hypertension, and dyslipidemia, are important risk factors for development of VDs.^2^ These risk factors have contributed to increased prevalence of VDs in the elderly population (> 60 years), with social and medical consequences.^3^ Aging provokes a physiological process of stiffening of the walls of blood vessels, increasing peripheral vascular resistance and reducing the supply of oxygen and nutrients to tissues.^1^ In conjunction with the endothelial oxidative stress provoked by diabetes mellitus (DM) and the chronic inflammation caused by deposition of lipids in dyslipidemia, this process creates the conditions that predispose to VDs. It has been estimated that from the age of 40 onwards, the risk of peripheral arterial occlusive disease increases two to three times with each additional 10 years of age.^2^ Occurrence of venous ulcers also increases with age and exceeds 4% in people over the age of 65.^4^

Vascular diseases are associated with important sequelae and clinical repercussions for the lives of affected patients, which are more serious among the elderly. The consequences of vascular disease, such as limb loss, chronic pain, prolonged hospitalization, and polypharmacy, erode these patients’ autonomy and independence, impacting their wellbeing and quality of life. The concept of quality of life is wide-ranging and should be considered on a case-by-case basis, encompassing physical health, psychological condition, self-esteem, and socioeconomic and cultural conditions, and for the elderly it should also incorporate the person’s degree of independence, showing that functional capacity is a parameter of health and changes the elderly person’s self-image and their conception of their role in society, causing feelings of fear, anguish, sadness and loneliness.^5^

Depression is common in the elderly population, with prevalence ranging from 4 to 23%,^6-9^ increasing among those with chronic diseases.^5^ Untreated depression in elderly people causes significant suffering and is linked to a five times greater chance of developing dementia over 3 years.^10^

In view of the above, recognition of the signs and symptoms of depressive disorder is essential to achieve early diagnosis and change these patients’ prognosis. The objective of this study is therefore to determine the prevalence of occurrence of depression and assess functional capacity in patients with vascular diseases admitted to a vascular surgery service.

## METHODS

This is a cross-sectional, prospective, observational study conducted at the vascular surgery service of a tertiary hospital, with a non-random sample selected consecutively from November 2018 to April 2019. A total of 127 patients aged 60 years or over with a diagnosis of vascular disease who signed a free and informed consent form were recruited consecutively. Four elderly patients with a prior diagnosis of dementia were excluded.

The data collection instruments employed were a sociodemographic questionnaire, a functional assessment scale, the Katz scale, and the Geriatric Depression Scale - Short Form (GDS-15).

The sociodemographic questionnaire was developed by the researchers and covered information to characterize the patients, such as sex, age, origin, educational level, and marital status. It also covered questions on lifestyle habits (smoking and alcoholism), use of medications, and information related to vascular diseases, such as lower limb ulcers, amputations, and pain.

The Geriatric Depression Scale is a 15-item questionnaire with objective responses about how the elderly respondent felt during the previous week. A GDS short form is a useful rapid assessment tool that facilitates identification of depression in the elderly. A GDS-15 cutoff point of 5/6 (not case/case) yields sensitivity of 85.4% and specificity of 73.9% for diagnosis of major depressive episodes according to the ICD-10. Scores range from 0 to 15, where scores from 0 to 5 are considered normal, from 6 to 10 are considered mild depression, and from 11 to 15 are considered severe depression.^11^

The Katz scale for functional assessment is designed to classify elderly patients according to their degree of dependence for performing daily activities of living (DAL), which are related to their capacity for self-care. Six functions are analyzed: bathing, dressing, toileting, transferring, continence, and feeding. The scores are summed to produce a final score, where 0 indicates a totally independent patient and 6 a totally dependent patient.^12,13^

The data collected were described as simple frequencies and percentages for categorical variables or means and standard deviations for continuous, ordinal, or discrete data. Associations between categorical variables were identified with Fisher’s exact test. The significance level was set at 5% and R Core Team 2017 software was used for analyses.

This project was approved by the Research Ethics Committee at the Universidade Federal de Sergipe, under protocol number CAAE: 48580115.2.0000.5546 and decision number 1.217.875.

Fisher’s exact test was used to test the study hypotheses and to determine the sample size. However, there is no well-formed formula to calculate sample sizes for Fisher’s exact test, so we used G*Power for this. Assuming a two-tailed test with 5% significance, 80% test power, 50% Group 1 proportion and 25% Group 2 proportion, with a 3:2 proportion between groups 2, 128 patients would be needed. The study recruited 127 participants, which we considered an adequate sample size.

## RESULTS

A total of 127 patients took part, 54.3% of whom were male and 45.7% of whom were female, with a mean age of 69.1 years. The majority had only attended primary education (63.8%). There was a predominance of retired individuals (80.3%), from provincial areas of the state of Sergipe (66.4%), who identified as Catholics (86.6%). Table 1 lists the sociodemographic characteristics of the patients in the study sample.

**Table 1:** Sociodemographic data.

	**N**	**%**
**Sex**		
Female	58	45.7
Male	69	54.3
**Origin**		
Capital	40	33.6
Interior	79	66.4
**Marital status**		
Single	31	24.4
Married/Stable partnership	65	51.2
Widowed	31	24.4
**Religion**		
Catholic	110	86.6
Evangelical	17	13.4
**Educational level**		
Illiterate	33	26.0
Primary education	81	63.8
Secondary education	13	10.2
**Employment status**		
Employed	25	19.7
Retired	102	80.3
**Monthly family income**		
< or =1 x MW	93	73.2
> 2 x MW	34	26.8

MW = minimum wage.

With relation to lifestyle habits, the majority were non-smokers (63.8%) and did not drink (68.5%), but just 15.7% engaged in physical activity regularly. Analysis of comorbidities and their frequencies revealed that 78% of subjects had systemic arterial hypertension (SAH) and 21.4% had DM. Table 2 lists the frequencies of comorbidities and also the drug classes used to treat these individuals, 79.5% of whom stated they had attended a consultation with a physician on the Family Health Strategy Program (FHSP) during the previous 12 months.

**Table 2:** Prevalence of comorbidities assessed and drug classes prescribed.

**Comorbidities**	**Total N (%)**
**SAH**	99 (78%)
ACEI	53 (41.7%)
ARA	58 (45.7%)
Diuretic	18 (14.2%)
Calcium channel blockers	10 (7.9%)
β-blockers	9 (7.1%)
α2-agonist	3 (2.4%)
**DM**	27 (21.4%)
Metformin	42 (33.1%)
Sulfonylurea	51 (40.2%)
Insulin NPH	3 (2.4%)
DPP4 inhibitor (Galvus)	3 (3.1%)

SAH = systemic arterial hypertension; ACEI = angiotensin-converting enzyme inhibitor; ARA = angiotensin II receptor antagonist; DM = diabetes mellitus; NPH = Neutral Protamine Hegedorn; DPP4 = dipeptidyl peptidase-4.

Around 46.5% of the interviewees had undergone an amputation and 83.5% reported pain in the lower limbs, 60.6% of whom had intermittent claudication.

Study subjects were assessed with the GDS-15, on which those scoring 0 to 5 points were considered free from depression, those scoring 6 to 10 points had mild depression, and those scoring 11 to 15 points were defined as having severe depression.

A total of 60 patients (47.2%) had mild depression, 50 (39.4%) were free from depression, and 17 (13.4%) had severe depression.

There were statistically significant associations between depression and the following conditions related to vascular diseases: consultation with an FHSP physician during the previous 12 months, alcohol use, claudication, diabetes, and individuals who had undergone an amputation (Table 3).

**Table 3:** Variables that exhibited significant associations (p < 0.05) with prevalence of depression.

	**Geriatric depression**			***p*-value**
	**No depression N (%)**	**Depression N (%)**	**Severe depression N (%)**	
Claudication	23 (46)	41 (69.5)	13 (81.3)	0.012
Diabetes mellitus	35 (71.4)	47 (78.3)	17 (100)	0.029
Amputees	17 (34)	29 (48.3)	13(76.5)	0.010
Consultation at the FHSP during the previous 12 months	37 (74)	55 (91.7)	9 (56.3)	0.002
				
Alcohol use	20 (40)	11 (18.3)	1 (5.9)	0.004

Fisher’s exact test. FHSP = Family Health Strategy Program.

Individuals with mild depression exhibited a higher prevalence of consultation with an FHSP physician during the previous 12 months (91.7%), while in the groups who did not have depression and with severe depression the rates were 74% and 56.3%, respectively. Patients who used alcohol were more prevalent in the group without depression (40%) than in the groups with mild depression (18.3%) and severe depression (5.9%). With relation to diabetes, 100% of severe depression patients had diabetes, while 71.4% were free from depression and 78.3% had mild depression. Among patients who had undergone an amputation, 76.5% exhibited severe depression, 37.6% had mild depression, and 34% did not have depression.

The results for the functional assessment scale had no statistically significant associations with sex (p = 0.07), monthly family income (p = 0.371), smoking (p = 0.198), or systemic arterial hypertension (p = 0.075). Table 4 lists the significant associations between sociodemographic variables and subjects’ functional capacity according to the Katz index.

**Table 4:** Variables that exhibited significant association (p < 0.05) with Katz index degree of dependence.

	**INTERPRETATION**			
	**Independent N (%)**	**Partially dependent N (%)**	**Dependent N (%)**	***p*-value**
**Sociodemographic variables**				
Educational level (illiterate)	8 (15.4)	15 (26.8)	10 (58.8)	0.019
Employment status (employed)	16 (30.8)	9 (16.1)	0 (0)	0.010
Alcohol use	21 (40.4)	9 (16.1)	0 (0)	0.002
**Conditions related to VDs and admission**				
Ulcers	30 (57.7)	50 (89.3)	12 (70.6)	0.001
Accompanied to the hospital	44 (84.6)	54 (96.4)	17 (100)	0.050
Consultation with FHSP during the previous 12 months	41 (78.8)	49 (87.5)	9 (56.3)	0.025
Claudication	16 (30.8)	47 (85.5)	13 (81.3)	< 0.001
Amputee	17 (32.7)	31 (55.4)	10 (58.8)	0.033

Fisher’s exact test. VD = vascular disease; FHSP = Family Health Strategy Program.

With regard to educational level, there was a higher prevalence of illiterate subjects among those classed as dependent (58.8%), compared to those classified as partially dependent (26.8%) and independent (15.4%). There was an increase in the proportion of independent subjects among those who had attended primary education (73.1%) and an increase in those who had attended secondary education (11.5%). With regard to employment status, 100% of the dependent patients were retired, while 30.8% of the patients considered independent and 16.1% of those considered partially dependent were employed.

## DISCUSSION

The majority of the population investigated in the present study were male, retired, married, and had low educational level and low income. These findings agree with reports by Pinto et al.,^14^ Tavares et al.^15^ and Faria^16^ and Neves et al.^17^ from studies that were similar in terms of the sociodemographic profile of patients admitted for VDs.

The proportion of elderly people in the general population has increased since the last century and this process is a result of demographic, nutritional, and epidemiological transitions that are modifying the causes of morbidity and mortality from infectious and contagious diseases to chronic noncommunicable conditions.^18^ In this context, according to Silva and Nahas,^19^ peripheral vascular diseases have become the most prevalent, specifically in the over-50s age group. These authors emphasize that the factor that most contributes to emergence of VDs among the elderly is the natural aging process that provokes degeneration and calcification of the vascular system, very often leading to functional incapacity and negatively impacting quality of life.

In this regard, Duarte and Rego^20^ stress that depression among patients with VDs has become a public health problem because of its frequent association with chronic diseases, leading to a negative impact on quality of life and suicide risk.

The present study found prevalence of depression of up to 60.6%. This is an elevated prevalence compared to studies conducted in other places in Brazil, which reported prevalence rates of 50% in São Luís (MA), 46.51% in Maceió (AL), 41.4% in the Federal District (DF), 27.6% in Aracaju (SE), 22% in rural areas of Minas Gerais (MG), and 16.4% in Florianópolis (SC).^21-27^ However, it is similar to the rate found by Mota et al.^28^ who studied occurrence of depressive symptoms in elderly patients with peripheral arterial disease, finding a 61.8% rate of depression.

With regard to sex, there was no significant association with geriatric depression, despite published data showing that there is a higher prevalence of depression among women.^20,29^

It is important to emphasize the relevance of depression in this sample and its relationship with non-transmissible chronic diseases, such as arterial hypertension and DM. According to Moreira et al.,^30^ presence of psychiatric symptoms in conjunction with organic disease can have a devastating effect on a person’s physical health and can even interfere with compliance with drug therapy. According to Blazer et al.,^31^ Blazer,^32^ and Snowdon,^33^ chronic disease increases the risk of developing depression and, according to Moreira et al.,^30^ there is an important association between depression and DM, which is also in agreement with the data found in the present study. These authors also state that this association ranges from a direct impact on metabolic control to adaptative, educational, and socioeconomic factors, but it is not possible to conclude that there is a causal relationship between depressive symptoms and glycemic control.

With relation to amputees, Parkes^34^ states that patients who have undergone an amputation have significant feelings of mutilation. Gabarra and Crepaldi^35^ reported that anxiety, impaired functional capacity, and depression are evident among these patients, confirming the findings of the present study. Fitzpatrick^36^ states that the cause of an amputation has a direct impact on psychological coping and that vascular causes have a different, less abrupt impact than traumatic causes. Involvement of an interdisciplinary health care team is therefore indispensable for providing these patients with integral care, with the objective of reducing morbidity and intervening directly in the health-disease process.

The present study also deals with the importance of quality of life (QoL) in patients with VDs. For example, venous disease has a major impact on QoL, which, according to Leal,^37^ is undeniable, given the elevated prevalence, the indolent clinical course that induces underestimation of its severity, and the lack of a perfect correlation between symptomology and objective signs found during physical examination. With regard to functional assessment of elderly patients, one variable that had a statistically significant association was educational level. There was a relationship through which the lower the educational level, the greater the degree of dependence, which has been confirmed by Cruz et al.,^38^ who reported that patients with higher educational levels preserved more cognitive functions and, as a result, better functional capacity.

In relation to ulcers, the study showed that 89.3% (50) are partially dependent, showing that these elderly people’s functional capacity declines, making them more dependent, which agrees with the literature, that states that ulcers are associated with significant pain, loss of mobility, and chronic diseases.^4^

Celich and Galon^39^ and Vaz et al.,^40^ analyzed the relationship between chronic pain and its influence on activities of daily living in the elderly, demonstrating that pain limits the possibilities for an elderly person to maintain normal daily life, i.e., it reduces functional capacity, negatively impacting quality of life and impairing activities of daily living to a certain extent. These data are similar to those observed in the present study, in which 85.5% of patients were partially dependent according to the Katz scale.

Since this study has a cross-sectional design, it cannot seek explanations for the associations observed. In other words, the investigator is a mere spectator of the phenomena or facts, without undertaking any type of intervention that might change their natural course and/or outcome. To study these effects, longitudinal cohort studies can be conducted to investigate relationships between factors associated with reduction of functional capacity and depression in greater detail and, consequently, develop strategies for health promotion and disease prevention in this population.

Both depression and impaired functional capacity can affect the quality of life of patients with vascular diseases. It is therefore the responsibility of the FHSP team to initially identify cases of claudication, diabetes, and alcoholism and provide health care for these patients, in addition to conducting early interventions to eliminate risk factors, since this is an avoidable disease when there is good control of cardiovascular risk factors and it is for this reason that the present study is intended to alert vascular surgeons and angiologists to the need to monitor their patients more closely, especially those whose functional capacity reduces during the course of treatments for VDs, with psychiatric disorders, such as depression.

## CONCLUSIONS

The findings of this investigation reveal a high prevalence of depression among people admitted with peripheral vascular disease and an important reduction in functional capacity in some groups, such as individuals with low educational level, those who had lower limb pain, diabetics, and those who had undergone an amputation.
