# Supplementary material for: Occurrence of depression and assessment of functional capacity in patients with vascular diseases admitted to a Vascular Surgery Service
Source: J Vasc Bras. 2023 Dec 18;22:e20230082. doi: 10.1590/1677-5449.202300821 (PMC10755889; doi:10.1590/1677-5449.202300821)
Supplement: Translation

# Translation: Ocorrência de depressão e avaliação da capacidade funcional em pacientes com doenças vasculares internados em um serviço de cirurgia vascular

**DOI:** 10.1590/1677-5449.202300822

**Authors:** José Aderval Aragão, Osmar Max Gonçalves Neves, Iapunira Catarina Sant’Anna Aragão, Felipe Matheus Sant’Anna Aragão, Bárbara Costa Lourenço, Luciano Conceição Porto, Pedro Henrique Adário Marassi, Francisco Prado Reis

**Affiliations:** 1 Universidade Federal de Sergipe - UFS, Aracaju, SE, Brasil.; 2 Fundação Beneficência Hospital Cirurgia - FBHC, Serviço de Cirurgia Vascular, Aracaju, SE, Brasil.; 3 Hospital Municipal Munir Rafful - HMMR, Volta Redonda, RJ, Brasil.; 4 Faculdade de Medicina de São José do Rio Preto - FAMERP, São José do Rio Preto, SP, Brasil.; 5 Faculdade de Ciências Médicas de Três Rios - FCM-TR, Três Rios, RJ, Brasil.; 6 Centro Universitário Tiradentes - UNIT, Maceió, AL, Brasil.; 7 Hospital Naval Marcílio Dias - HNMD, Rio de Janeiro, RJ, Brasil.; 8 Universidade Tiradentes - UNIT, Aracaju, SE, Brasil.; 9 Centro Universitário Alfredo Nasser - UNIFAN, Aparecida de Goiânia, GO, Brasil.

**Keywords:** depressão vascular, doenças vasculares, testes de aptidão, idoso fragilizado, atividades cotidianas

## Abstract

**Contexto:**

As doenças vasculares estão associadas a importantes sequelas e repercussões clínicas nas vidas dos pacientes acometidos e, em maior relevância, entre os idosos. Consequências da doença vascular como a perda de um membro, dor crônica, internamentos prolongados e a polifarmácia geram, nesses pacientes, perda de autonomia e um grau de dependência, que vão influenciar o bem-estar e a qualidade de vida.

**Objetivos:**

Determinar a ocorrência de depressão e avaliar a capacidade funcional em pacientes com doenças vasculares internados em um serviço de cirurgia vascular.

**Métodos:**

Trata-se de um estudo observacional, transversal, prospectivo, realizado no serviço de cirurgia vascular de um hospital terciário, com uma amostra não aleatória selecionada de forma consecutiva. Para avaliar a depressão, foi utilizada a escala de depressão geriátrica resumida e, para a avaliação funcional do indivíduo, foi utilizada a escala de Katz.

**Resultados:**

A prevalência de depressão nesses pacientes foi de 60,6%. Foi observada associação entre depressão e: consulta com médico do Programa de Estratégia de Saúde da Família nos últimos 12 meses, etilismo, claudicação, diabetes e indivíduos que sofreram amputação. Já em relação à capacidade funcional do indivíduo avaliado através do índice de Katz, ocorreram associações significativas entre variáveis sociodemográficas, condições relacionadas a doença vascular e internamento.

**Conclusões:**

Existem uma alta prevalência de depressão em pacientes com doenças vasculares internados em um serviço de cirurgia vascular e uma redução importante da capacidade funcional em alguns grupos, como os indivíduos de baixa escolaridade, os que sentiam dor crônica nos membros inferiores, os diabéticos e aqueles que sofreram amputação.

## INTRODUÇÃO

As doenças vasculares (DVs) são resultado de alterações fisiopatológicas no sistema arterial, venoso ou linfático, como a redução do fluxo sanguíneo comprometendo o aporte de nutrientes e gerando isquemia ou estase vascular, a depender do vaso acometido e da demanda tecidual. O acometimento arterial provoca manifestações clínicas, como dor, alterações tróficas e de coloração, resfriamento do membro e rarefação de pelos. No caso de acometimento venoso, estão presentes edema, hiperpigmentação, hiperidrose, eczema, úlceras e dermatofibrose. Em uma afecção linfática, a dor e o edema são mais significantes^1^ . As doenças crônicas não transmissíveis, como o diabetes, hipertensão arterial e dislipidemia, são importantes fatores de risco para o desenvolvimento das DVs^2^ . Esses fatores de risco têm contribuído para o aumento da prevalência das DVs na população de idosos (> 60 anos), gerando consequências sociais e médicas^3^ . O envelhecimento provoca um processo fisiológico de enrijecimento da parede dos vasos sanguíneos, aumentando, assim, a resistência vascular periférica e diminuindo o aporte de oxigênio e nutrientes aos tecidos^1^ . Esse processo, associado ao estresse oxidativo do endotélio provocado pelo diabetes mellitus (DM) e à inflamação crônica causada pela deposição de lipídios na dislipidemia, gera condições que predispõem às DVs. Tem sido estimado que, após os 40 anos, o risco da doença arterial obstrutiva periférica aumenta de duas a três vezes a cada incremento de 10 anos na idade^2^ . A ocorrência de úlcera venosa aumentaria com a idade e seria superior a 4% em pessoas acima dos 65 anos de idade^4^ .

As DVs estão associadas a importantes sequelas e repercussões clínicas nas vidas dos pacientes afetados, com maior relevância entre os idosos. Consequências da doença vascular, como perda de um membro, dor crônica, internamentos prolongados e polifarmácia geram, nesses pacientes, perda de autonomia e um grau de dependência que vão influenciar o bem-estar e a qualidade de vida. O conceito de qualidade de vida é amplo e deve ser considerado de maneira individualizada, abrangendo saúde física, condição psicológica, autoestima, condições socioeconômicas e culturais e, na faixa etária dos idosos, deve incorporar o grau de independência do indivíduo, evidenciando a capacidade funcional como um parâmetro de saúde, alterando a concepção do idoso sobre si mesmo e sobre o seu papel na sociedade, gerando sentimentos de medo, angústia, tristeza e solidão^5^ .

A depressão na população idosa é frequente, com prevalência que varia de 4 a 23%^6-9^ , sendo ainda maior nos portadores de doenças crônicas^5^ . A depressão não tratada em idosos causa sofrimento significativo e tem até cinco vezes mais chances de desenvolver demência ao longo de três anos^10^ .

Nesse contexto, é fundamental para um diagnóstico precoce e alteração do prognóstico desses pacientes o reconhecimento dos sinais e sintomas do transtorno depressivo. Dessa maneira, o presente estudo tem como objetivo determinar a ocorrência de depressão e avaliar a capacidade funcional em pacientes com doenças vasculares internados em um serviço de cirurgia vascular.

## MÉTODOS

Trata-se de um estudo observacional de corte transversal, prospectivo, realizado no serviço de cirurgia vascular de um hospital terciário, com uma amostra não aleatória selecionada de forma consecutiva durante o período de novembro de 2018 a abril de 2019. Foram incluídos, consecutivamente, 127 pacientes com idade igual ou superior a 60 anos, com diagnóstico de patologias vasculares e que assinaram o termo de consentimento livre e esclarecido. Foram excluídos quatro idosos com diagnóstico prévio de demência.

Para a coleta de dados, foram utilizados como instrumentos: um questionário sociodemográfico, escala para avaliação funcional do indivíduo, ou escala de Katz, e a escala de depressão geriátrica resumida (EDG-15).

O questionário sociodemográfico foi confeccionado pelos pesquisadores e constava de informações para melhor caracterização dos pacientes, como sexo, idade, procedência, escolaridade e estado civil. Além de questões relativas a hábitos de vida (tabagismo e etilismo), uso de medicações e informações relacionadas a doenças vasculares, como presença de feridas, amputações e dor em membros inferiores.

A escala de depressão geriátrica (EDG) é um questionário com 15 perguntas e respostas objetivas a respeito de como o idoso se sentiu durante a semana anterior. A EDG abreviada é uma ferramenta útil de avaliação rápida para facilitar a identificação da depressão em idosos. O ponto de corte 5/6 (não caso/caso) para a EDG-15 produziu índices de sensibilidade de 85,4% e especificidade de 73,9% para o diagnóstico de episódio depressivo maior, de acordo com o CID-10. A pontuação varia de 0 a 15, sendo a pontuação de 0 a 5 considerada normal, a de 6 a 10 considerada depressão leve e a de 11 a 15 considerada depressão severa^11^ .

A escala para avaliação funcional do indivíduo, ou escala de Katz, busca classificar o idoso quanto ao seu grau de dependência ao executar as atividades de vida diária (AVDI), as quais são referentes à capacidade de autocuidado. São analisadas seis funções: banhar-se, vestir-se, ir ao banheiro, transferência, continência e alimentação. A partir de pontuações atingidas, define-se o escore final, no qual 0 é o idoso totalmente independente e a pontuação de 6 é aquele totalmente dependente^12,13^ .

Os dados coletados foram descritos por meio de frequência simples e percentuais quando categóricos ou média e desvio padrão quando contínuos, ordinais ou discretos. Para avaliar a associação entre as variáveis categóricas, foi utilizado o teste Exato de Fisher. O nível de significância adotado foi de 5%, e o *software* utilizado foi o R Core Team 2017.

Este projeto foi aprovado pelo Comitê de Ética em Pesquisa da Universidade Federal de Sergipe, com o protocolo nº CAAE: 48580115.2.0000.5546 e número do parecer 1.217.875.

Para testar as hipóteses deste estudo e determinar o tamanho da amostra, foi utilizado o teste exato de Fisher. Contudo, não há fórmula fechada para calcular tamanhos amostrais para o teste exato de Fisher, então, usamos o software G*Power para isso. Assumindo um teste bicaudal com significância de 5%, poder de teste de 80%, proporção do Grupo 1 de 50% e do Grupo 2 de 25%, com uma proporção entre os grupos de 3:2, são necessários 128 pacientes. Este estudo obteve 127 participantes, donde consideramos um tamanho amostral adequado.

## RESULTADOS

Participaram 127 pacientes, 54,3% do sexo masculino e 45,7% do sexo feminino, com idade média de 69,1 anos. A maioria frequentou somente o ensino fundamental (63,8%). O predomínio foi de indivíduos aposentados (80,3%), procedentes do interior de Sergipe (66,4%) e de católicos (86,6%). A Tabela 1 especifica as características sociodemográficas dos pacientes sujeitos da pesquisa.

**Tabela 1:** Dados sociodemográficos.

	**N**	**%**
**Sexo**		
Feminino	58	45,7
Masculino	69	54,3
**Procedência**		
Capital	40	33,6
Interior	79	66,4
**Estado civil**		
Solteiro	31	24,4
Casado/União estável	65	51,2
Viúvo	31	24,4
**Afiliação religiosa**		
Católico	110	86,6
Evangélico	17	13,4
**Escolaridade**		
Analfabeto	33	26,0
Fundamental	81	63,8
Médio	13	10,2
**Estado ocupacional**		
Empregado	25	19,7
Aposentado	102	80,3
**Renda familiar mensal**		
< ou =1 SM	93	73,2
> 2 SM	34	26,8

SM = salário mínimo.

Com relação aos hábitos de vida, a maioria não era tabagista (63,8%) nem etilista (68,5%), e apenas 15,7% praticavam atividade física regular. Na avaliação das comorbidades e sua frequência, 78% dos indivíduos tinham hipertensão arterial sistêmica (HAS) e 21,4% DM. A Tabela 2, além da frequência das comorbidades, demonstra as classes medicamentosas utilizadas no tratamento desses indivíduos, dos quais 79,5% afirmaram ter feito consulta com médico do Programa de Estratégia de Saúde da Família (PESF) nos últimos 12 meses.

**Tabela 2:** Prevalência das comorbidades avaliadas e classes medicamentosas utilizadas.

**Comorbidades**	**Total N (%)**
**HAS**	99 (78%)
IECA	53 (41,7%)
BRA	58 (45,7%)
Diurético	18 (14,2%)
Bloqueador de canais de cálcio	10 (7,9%)
β-bloqueador	9 (7,1%)
α2-agonista	3 (2,4%)
**DM**	27 (21,4%)
Metformina	42 (33,1%)
Sulfonilureia	51 (40,2%)
Insulina NPH	3 (2,4%)
Inibidor de DPP4 (Galvus)	3 (3,1%)

HAS = hipertensão arterial sistêmica; IECA = inibidor da enzima conversora de angiotensina; BRA = antagonista dos receptores de angiotensina II; DM = diabetes mellitus; NPH = *Neutral Protamine Hegedorn*; DPP4 = dipeptidil peptidase-4.

Cerca de 46,5% dos indivíduos entrevistados sofreram amputação, e 83,5% referiram ter dor nos membros inferiores e, desses, 60,6% apresentavam claudicação intermitente.

Os indivíduos foram avaliados a partir da EDG-15, na qual indivíduos com pontuação de 0 a 5 foram considerados como ausência de depressão, de 6 a 10 pontos, com depressão leve, e com 11 a 15 pontos, com depressão grave.

Foram encontrados 60 pacientes (47,2%) com depressão leve, 50 (39,4%) sem depressão, e 17 (13,4%) apresentavam depressão grave.

A associação entre a depressão e condições relacionadas às doenças vasculares foram estatisticamente significantes em relação a: consulta com médico do PESF nos últimos 12 meses, etilistas, claudicação, diabetes e indivíduos que já sofreram amputação (Tabela 3).

**Tabela 3:** Variáveis que apresentaram associação significativa (p < 0,05) com a prevalência de depressão.

	**Depressão geriátrica**			
	**Sem depressão N (%)**	**Depressão N (%)**	**Depressão grave N (%)**	**p-valor**
				
Claudicação	23 (46)	41 (69,5)	13 (81,3)	0,012
				
Diabetes mellitus	35 (71,4)	47 (78,3)	17 (100)	0,029
Amputados	17 (34)	29 (48,3)	13(76,5)	0,010
Consulta no PESF nos últimos 12 meses	37 (74)	55 (91,7)	9 (56,3)	0,002
				
Etilista	20 (40)	11 (18,3)	1 (5,9)	0,004

Teste Exato de Fisher. PESF = Programa de Estratégia de Saúde da Família.

Os indivíduos com depressão leve apresentaram maior prevalência de consulta com o médico no PESF nos últimos 12 meses (91,7%) e, em relação aos grupos que não tinham depressão e com depressão grave, os resultados foram 74% e 56,3%, respectivamente. Os etilistas foram os mais prevalentes no grupo sem depressão (40%), contra 18,3% no grupo com depressão leve e 5,9%, com depressão grave. Com relação ao diabetes, 100% dos depressivos graves eram diabéticos, enquanto 71,4% não eram depressivos, e 78,3% tinham depressão leve. Nos pacientes que sofreram amputação, 76,5% apresentaram depressão grave, 37,6% depressão leve e 34% não tinham depressão.

No que diz respeito à escala para avaliação funcional do indivíduo, é importante salientar que não houve associação estatisticamente significante com sexo (p = 0,07), renda familiar mensal (p = 0,371), tabagismo (p = 0,198) e hipertensão arterial sistêmica (p = 0,075). A Tabela 4 demonstra as associações significativas entre as variáveis sociodemográficas e a capacidade funcional do indivíduo avaliada através do índice de Katz.

**Tabela 4:** Variáveis que apresentaram associação significativa (p < 0,05) com o grau de dependência do índice de Katz.

	**INTERPRETAÇÃO**			**p-valor**
	**Independente N (%)**	**Parcialmente dependente N (%)**	**Dependente N (%)**	
**Variáveis sociodemográficas**				
Escolaridade (analfabeto)	8 (15,4)	15 (26,8)	10 (58,8)	0,019
Estado ocupacional (empregado)	16 (30,8)	9 (16,1)	0 (0)	0,010
Etilismo	21 (40,4)	9 (16,1)	0 (0)	0,002
**Condições relacionadas a DVs e internamento**				
Ferida	30 (57,7)	50 (89,3)	12 (70,6)	0,001
Acompanhamento ao hospital	44 (84,6)	54 (96,4)	17 (100)	0,050
Consulta no PESF nos últimos 12 meses	41 (78,8)	49 (87,5)	9 (56,3)	0,025
Claudicação	16 (30,8)	47 (85,5)	13 (81,3)	< 0,001
Já realizou amputação	17 (32,7)	31 (55,4)	10 (58,8)	0,033

Teste Exato de Fisher. DV = doença vascular; PESF = Programa de Estratégia de Saúde da Família.

Quanto à escolaridade, houve uma maior prevalência de analfabetos entre os considerados dependentes (58,8%) em relação aos parcialmente dependentes (26,8%) e aos independentes (15,4%). Houve um aumento do grupo dos independentes naqueles que frequentaram o ensino fundamental (73,1%) e um aumento quando se tratou dos que frequentaram o ensino médio (11,5%). Sobre o estado ocupacional, 100% dos pacientes dependentes eram aposentados, 30,8% dos pacientes considerados independentes estavam empregados, assim como 16,1% dos parcialmente dependentes.

## DISCUSSÃO

A população da presente investigação foi composta na sua maioria por homens, aposentados, casados e que apresentavam uma baixa escolaridade e renda. Esses achados concordam com o relatado por Pinto et al.^14^ , Tavares et al.^15^ e Faria^16^ e Neves et al.^17^ em estudos semelhantes referentes ao perfil sociodemográfico de pacientes internados por conta de DVs.

A proporção de idosos na população geral tem aumentado desde o último século, e esse processo é decorrente das transições demográfica, nutricional e epidemiológica, modificando as causas de morbimortalidade, de doenças infectocontagiosas para as crônicas não transmissíveis^18^ . Nesse contexto, para Silva e Nahas^19^ , as doenças vasculares periféricas têm sido mais prevalentes, em especial, na faixa etária acima dos 50 anos de idade. Os autores destacaram que o fator que contribui, entre os idosos, para o surgimento da DVs seria o processo natural de envelhecimento humano que promove degeneração e calcificação do sistema vascular, levando, muitas vezes, à incapacidade funcional e influenciando negativamente na sua qualidade de vida.

Nesse contexto, Duarte e Rego^20^ enfatizaram que a depressão entre os pacientes portadores de DVs se tornou um problema de saúde pública em decorrência ainda de sua frequente associação com doenças crônicas, o que levaria a um impacto negativo na qualidade de vida e ao risco de suicídio.

No presente estudo, foi encontrada uma prevalência de depressão de 60,6%. Trata-se de uma prevalência elevada quando comparada a estudos nacionais conduzidos em diferentes locais do Brasil, os quais apresentaram prevalências de 50% em São Luís (MA), 46,51% em Maceió (AL), 41,4% no Distrito Federal (DF), 27,6% em Aracaju (SE), 22% na zona rural de Minas Gerais (MG) e 16,4% em Florianópolis (SC)^21-27^ . Porém, é semelhante ao encontrado por Mota et al.^28^ que, estudando a ocorrência de sintomas depressivos em paciente idosos com doença arterial periférica, encontrou uma taxa de depressão de 61,8%.

Com relação ao sexo, não houve associação significativa com a depressão geriátrica, apesar de os dados da literatura afirmarem que há uma maior prevalência de depressão em mulheres^20,29^ .

É importante salientar a relevância da presença de depressão nessa amostra e a sua relação com doenças crônicas não transmissíveis, como hipertensão arterial e DM. De acordo com Moreira et al.^30^ , a presença de sintomas psiquiátricos associados a uma doença orgânica pode ter um efeito devastador sobre a saúde física do indivíduo, podendo interferir inclusive na adesão à terapêutica medicamentosa. Para Blazer et al.^31^ , Blazer^32^ e Snowdon^33^ , uma doença crônica aumenta o risco de desenvolver depressão; e para Moreira et al.^30^ , há uma importante associação entre depressão e DM, o que também corrobora os dados encontrados em nosso estudo. Esses autores afirmaram, ainda, que essa associação abrange desde o impacto direto no controle metabólico até aspectos adaptativos, educacionais e socioeconômicos, entretanto, não é possível afirmar a existência de uma relação causal entre os sintomas depressivos e o controle glicêmico.

Com relação aos indivíduos amputados, Parkes^34^ afirmou que há um sentimento de mutilação importante em pacientes que sofreram amputação. Gabarra e Crepaldi^35^ afirmaram que a presença de ansiedade, diminuição da capacidade funcional e depressão tornaram-se evidentes nesses pacientes, o que confirma os achados do presente estudo. Fitzpatrick^36^ relata que a causa da amputação interfere diretamente no enfrentamento psicológico e que causas vasculares possuem um impacto diferente e menos brusco comparadas a causas traumáticas. Diante desse cenário, é imprescindível a atuação de uma equipe de saúde interdisciplinar no cuidado integral a esses pacientes, com o objetivo de reduzir a morbidade e influenciar diretamente nos condicionantes do processo saúde-doença.

O presente estudo tratou também da importância da qualidade vida (QV) em pacientes com DVs. A doença venosa, por exemplo, tem grande impacto na QV, o que, para Leal^37^ , é indiscutível devido à elevada prevalência, ao curso clínico indolente conduzindo à subestimativa da sua severidade, à ausência de correlação perfeita entre a sintomatologia e aos sinais objetivos ao exame físico do doente. Quanto à avaliação funcional do idoso, a variável que apresentou significância estatística foi a escolaridade. Foi estabelecida a relação de que quanto menor a escolaridade, maior o grau de dependência, o que é confirmado por Cruz et al.^38^ quando afirmaram que aqueles pacientes com maior grau de escolaridade mantiveram mais funções cognitivas e, com isso, melhor grau de capacidade funcional.

Em relação a feridas, este estudo mostrou que 89,3% (50) são parcialmente dependentes, atestando que há um decréscimo da capacidade funcional do idoso, tornando-o mais dependente, o que está concordante com a literatura, que afirma que feridas se associam a dores importantes, perda de mobilidade e doenças crônicas^4^ .

Celich e Galon^39^ e Vaz et al.^40^ , analisando a relação da dor crônica em idosos e sua influência nas atividades de vidas diárias, demostraram que a dor limita a possibilidade do idoso em manter seu cotidiano de maneira normal, ou seja, diminuindo a capacidade funcional, o que impacta negativamente sua qualidade de vida, prejudicando, de algum modo, a realização das atividades de vida diária. Esses dados são semelhantes aos do presente estudo, em que 85,5% dos pacientes, de acordo com a escala de Katz, eram parcialmente dependentes.

Este estudo, por ter um desenho metodológico transversal, não teve a incumbência de buscar explicações para as associações encontradas, ou seja, o investigador atua meramente como expectador de fenômenos ou fatos, sem, no entanto, realizar qualquer intervenção que possa interferir no curso natural e/ou no desfecho dos mesmos. Por isso, é necessário que estudos longitudinais do tipo coorte possam ser realizados para investigar mais detalhadamente as relações entre os fatores associados à redução da capacidade funcional e depressão e, consequentemente, desenvolver estratégias de promoção da saúde e prevenção de doenças nessa população.

Por isso, a depressão, bem como a diminuição da capacidade funcional, pode influenciar na qualidade de vida dos pacientes com doenças vasculares. Nesse sentido, inicialmente, é dever das equipes do PESF identificar os casos de claudicação, diabetes, etilismo e promover a atenção à saúde dos mesmos, além da intervenção precoce para a eliminação de fatores de risco, já que é uma doença evitável quando há um bom controle dos fatores de risco cardiovasculares. E é nesse sentido que o presente estudo pretende despertar para os cirurgiões vasculares e angiologistas um olhar mais vigilante a pacientes, em especial, àqueles que são acometidos de redução da capacidade funcional no curso de tratamentos de DVs de transtornos psíquicos, como depressão.

## CONCLUSÃO

Com os achados desta investigação, constataram-se uma alta prevalência de depressão entre os indivíduos internados com doença vascular periférica e uma redução importante da capacidade funcional em alguns grupos, como os indivíduos de baixa escolaridade, os que sentiam dor nos membros inferiores, os diabéticos e aqueles que sofreram amputação.
